# Multiple Targets CFAR Detection Performance Based on an Intelligent Clustering Algorithm in K-Distribution Sea Clutter

**DOI:** 10.3390/s25082613

**Published:** 2025-04-20

**Authors:** Mansoor M. Al-dabaa, Eugen Laslo, Ahmed A. Emran, Ahmed Yahya, Ashraf Aboshosha

**Affiliations:** 1 Department of Electrical Engineering, Faculty of Engineering, Al-Azhar University, Cairo 11651, Egypt; ahmed.emran@azhar.edu.eg (A.A.E.); dr.ahmed.yahya@azhar.edu.eg (A.Y.); 2 Department of Mathematics and Computer Science, Faculty of Science, University of Oradea, 410087 Oradea, Romania; 3 Radiation Engineering Department, National Center for Radiation Research and Technology (NCRRT), Egyptian Atomic Energy Authority (EAEA), Cairo 11371, Egypt; ashraf.aboshosha@eaea.sci.eg

**Keywords:** K-distribution sea clutter, linear density-based spatial clustering for applications with noise, cell under test, sea clutter, multiple targets

## Abstract

Maintaining a Constant False Alarm Rate (CFAR) in the presence of K-distributed sea clutter is vital due to the dynamic and unpredictable nature of maritime environments. However, conventional CFAR detectors suffer significant performance degradation in multi-target scenarios, primarily due to the masking effect caused by interfering targets. To address this challenge, this paper introduces an advanced detection scheme that integrates Linear Density-Based Spatial Clustering for Applications with Noise (Lin-DBSCAN) with CFAR processing. Lin-DBSCAN is specifically tailored to efficiently identify and isolate interfering targets and sea spikes, which typically manifest as outliers in the symmetric reference windows surrounding the Cell Under Test (CUT). By leveraging Lin-DBSCAN, the proposed Lin-DBSCAN-CFAR method effectively filters out anomalous signals from the background clutter, resulting in enhanced detection accuracy and robustness, especially under complex sea clutter conditions. Extensive simulations under varying conditions, including multiple target environments, varying false alarm rates, and different clutter shape parameters, demonstrate that Lin-DBSCAN-CFAR significantly outperforms conventional CFAR approaches. It is noteworthy that the proposed method achieves detection performance comparable to the more computationally intensive DBSCAN-CFAR while significantly reducing computational complexity. Simulation results reveal that Lin-DBSCAN-CFAR requires a 1 to 2 dB lower SNR to reach a detection probability of 0.8 compared with the nearest traditional CFAR techniques, confirming its superiority in both accuracy and efficiency.

## 1. Introduction

To accurately model and simulate sea clutter at high resolution, the K distribution was introduced as a reliable alternative [1]. This choice was made due to the limitations of conventional statistical distributions such as Rayleigh, Weibull, and lognormal in effectively describing the statistical characteristics of sea clutter [2]. In the K distribution model, the amplitude of sea clutter is assumed to follow the Rayleigh distribution (referred to as the speckle component) at each distance unit, while the intensity is governed by the gamma distribution. The effectiveness of the K distribution model in capturing the clutter scattering mechanism has been rigorously tested and evaluated in real-world applications, utilizing experimental data. These evaluations have demonstrated the efficiency and reliability of the K distribution model in accurately representing the complex characteristics of clutter, further validating its practical applicability in various real-world scenarios [3,4].

The primary purpose of a radar system is to detect targets. CFAR processors, as an efficient method of primary target identification, can increase the probability of detection while maintaining a constant false alarm rate [5]. Previous studies have presented various CFAR processors based on the sliding reference window technique. To accomplish the goal of automatic target detection, these processors employ a statistical analysis of the clutter background. Drawing from a significant body of research findings [6,7], the process involves the computation of dynamic thresholds that are subsequently juxtaposed against the values derived from the CUT. This methodology enables the comparison and evaluation of these values, allowing for the effective discrimination and identification of targets in diverse scenarios [8,9].

The best-known processors are the mean-level processors, which include the smallest-of CFAR (SO-CFAR) [10], average-cell CFAR (CA-CFAR) [11], and greatest-of CFAR (GO-CFAR) [12]. However, the best detection performance may not be achieved when used in a complicated background, such as K-distribution marine clutter, especially when multiple targets are present. When one or more interfering targets are present within the reference window, the strong target masking effect can lead to a higher probability of missing targets [13,14]. Consequently, to limit this negative effect, a more precise assessment approach for the background noise level has been developed. As a result, a class of classification-based processors, such as CFAR Ordered Statistics, is required in addition to mid-level processors (OS-CFAR). In OS-CFAR [10], the sampled values within the reference window are first sorted. Subsequently, a specific reference cell is selected based on the desired range. To estimate the average power of the clutter, a predefined or excluded portion of the reference cells with the highest amplitudes is utilized. When estimating the background level, sea spikes and interfering targets within the reference window will be considered outliers. In multi-target scenarios, sorting-based processors outperform SO, GO, and CA-CFAR, although they depend on prior knowledge about the distribution and number of interference targets [7].

Over the past few years, deep learning has demonstrated remarkable effectiveness in tackling the challenge of noisy labels in image classification. Notably, the study titled PSSCL: A Progressive Sample Selection Framework with Contrastive Loss for Learning with Noisy Labels proposes a dynamic framework that incrementally selects cleaner samples and leverages contrastive loss, ultimately yielding more robust learning outcomes [15]. Meanwhile, BPT-PLR: Balanced Partitioning and Training with Pseudo-Label Relaxed Contrastive Loss for Noisy Label Learning introduces a method that harmonizes data partitioning and model training by employing a relaxed contrastive loss based on pseudo-labels, thereby enhancing resilience against mislabeled data [16]. Furthermore, the paper Cross-to-Merge Training with Class Balance Strategy for Learning with Noisy Labels presents a strategy that merges cross-sample knowledge and maintains class balance, thus further strengthening learning under noisy conditions [17].

Recent research underscores the critical hurdle of interference in multichannel adaptive signal detection, especially subspace interference, profoundly impacting the accuracy of target detection amidst multiple threats. Addressing this challenge, advanced CFAR techniques have emerged, meticulously tailored to mitigate interference’s adverse effects. Notably, the Interference Cancellation Before Detection (ICBD) method stands out as the linchpin in this approach, prominently featured in [18], adeptly managing interference within training data to notably augment adaptive detectors’ capabilities.

Furthermore, Refs. [19,20] amplifies the significance of this method by advocating for sophisticated detector designs adept at navigating interference complexities. These designs are crucial for ensuring robust and effective detection in scenarios riddled with such challenges.

Machine learning has attracted a lot of interest in recent years as a multidisciplinary subject. Clustering algorithms, artificial neural networks (ANN), and deep learning [21], among other machine learning technologies, have been applied to various academic topics. Clustering algorithms, such as DBSCAN (Density-Based Spatial Clustering of Applications with Noise), LOF (Local Outlier Factor), k-means, and isolation forests, play a crucial role in machine learning. These tools are widely used for tasks like grouping data points, detecting anomalies, and identifying patterns within datasets [13,22,23,24]. Clustering algorithms are well-suited to recognize radar targets in non-uniform jamming backgrounds. In the context of multi-target scenarios, a revised CA-CFAR method based on the Grubbs criterion has been documented in [25]. Nonetheless, it is essential to note that in a univariate dataset exhibiting an approximately normal distribution, Grubbs’ criterion solely serves as an effective tool for identifying a single outlier. It cannot be used to produce the best detection results for a K-distribution disorder due to this limitation. Research utilizing clustering techniques to address the challenge of multi-target CFAR detection in marine environments with K-distributed interference remains limited [26,27].

The proposition of utilizing DBSCAN clustering [13] as the backbone for an advanced CFAR processor represents a leap in efficiency and robustness compared with conventional models, albeit with added complexity and cost implications. The computational overhead associated with this technique scales proportionally with the dataset size, despite attempts to improve execution time within the confines of O(n log n) complexity. To confront these challenges head-on, our focus pivots toward developing a novel CFAR processor harnessing the power of Lin-DBSCAN clustering technology. Lin-DBSCAN, purpose-built to surmount DBSCAN’s computational hurdles with smaller datasets while preserving sequential programming principles, amalgamates density properties and grid-based clustering. This transformative approach diverges from scrutinizing individual points to directly evaluating grid cells, promising more streamlined data processing strategies.

The core innovation of our proposed method lies in integrating the Lin-DBSCANa linear-time clustering algorithm into the CFAR detection framework specifically tailored for K-distributed sea clutter in maritime environments. Unlike conventional CFAR techniques, which often rely on fixed clutter assumptions or extensive prior knowledge of interference distributions, Lin-DBSCAN adaptively identifies and excludes sea spikes and overlapping targets by employing a grid-based discretization of the surrounding reference cells. This approach bypasses the exhaustive pairwise distance calculations required by traditional DBSCAN, resulting in substantially lower computational overhead. By relying on minimal hard-coded thresholds, our method dynamically adjusts to evolving clutter conditions, thereby enhancing multi-target detection capabilities and reducing reliance on strict, static models.

At the end of the introduction, we summarize our paper’s key contributions, emphasizing the innovative aspects and methodological advancements of our proposed approach. This work is distinguished by the following core contributions:

- We propose the Lin-DBSCAN-CFAR processor, a novel CFAR detection method that incorporates Lin-DBSCAN clustering to adaptively suppress anomalies and outliers near the Cell Under Test (CUT) without requiring prior knowledge of interference target numbers or distributions.
- The main contribution of this work is the significant reduction in computational complexity compared with traditional DBSCAN, achieved by applying the proposed Lin-DBSCAN-CFAR approach. This enables faster and more efficient performance in real-time applications.
- We address the limitations of DBSCAN-CFAR by achieving comparable detection accuracy with a significantly lower computational cost, offering a practical balance between robustness and efficiency.
- We demonstrate that grid-level analysis, rather than individual point analysis, enables streamlined and scalable clutter estimation, enhancing detection reliability in complex, multi-target maritime scenarios.

This is the main structure of this paper: The Section 2 explains the concepts of the K-distribution model of the sea clutter and detection by CFAR. The implementation details of Lin-DBSCAN-CFAR are discussed in depth in the Section 3. The Section 4 focuses on Lin-DBSCAN-CFAR processor evaluation and results. Finally, the Section 5 presents the important conclusions drawn from the study and outlines potential avenues for future exploration.

## 2. The Conceptual Framework of the K-Distribution Sea Clutter Model and CFAR Detection

### 2.1. K-Distribution Sea Clutter Model

The K-distribution represents an intricate model that possesses the remarkable ability to accurately depict echoes stemming from various sea surface states. The primary utility of this model lies in its application for analyzing the scattering radiation observed at low backscatter angles with utmost precision [3,4]. The probability density function (PDF) of a quantitative distribution is defined by the following expression:

(1) $$f\left(x\right)=\frac{4v}{\Gamma (v)}{\left(cv\right)}^{v}k\left(v-1\right)\left(2cx\right)$$

where *c* is the scaling parameter; it is the average sea clutter power. *v* is the shape parameter. Γ(v) is the gamma function. K(v−1) is the modified Bessel function [4].

### 2.2. CFAR Detection

The main goal of a CFAR procedure is to establish a flexible threshold that can effectively distinguish between target and radar jamming signals. This dynamic threshold is closely related to the probability of intended false alarms and the level of background noise. Its purpose is to accurately detect the presence of a target with a minimum incidence of false alarms caused by external interference [28].

After the combined filtering processes, the quadrupole and in-phase components of the complex radar signal are processed using the square law. The ensuing outputs are subsequently subjected to a sliding window configuration comprising the reference cells, the experimental CUT, and the guard reference cells, as elucidated in [29]. Suppose the reference cell samples are {x1,x2,…,xn} in the main window and {xn+1,xn+2,…,x2n} in the sliding window, where the length of the reference window is represented by N = 2n. For CUT, the leading and trailing windows are the same. The spatial and temporal correlation exhibited by the radar echo permits a reliable estimation of the noise background level by utilizing the reference cells surrounding the CUT, as supported by the findings in [12]. To reduce the impact of extended targets on detection performance, CFAR processors like GO, CA, and SO-CFAR typically include a few protection cells on either side of the CUT.

## 3. Lin-DBSCAN-CFAR Processor

In this specific section, the Lin-DBSCAN-CFAR process is used to obtain an accurate estimate of the background level associated with sea surface clutter. This estimation is achieved by implementing an ANN model, which accurately evaluates the shape parameter of sea surface clutter.

**Figure 1:**
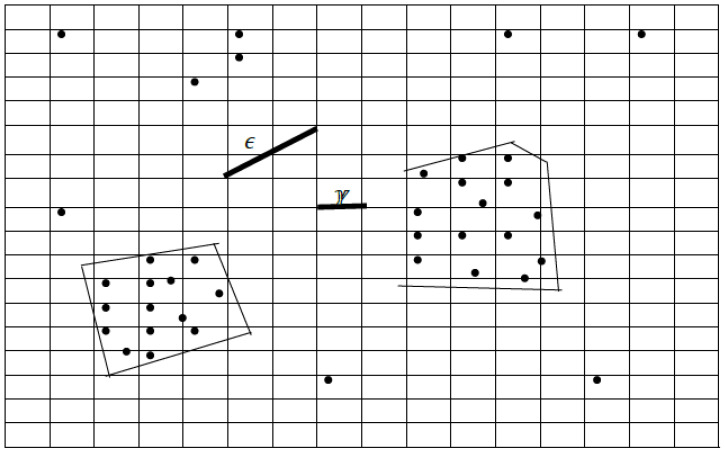
Relationship between ϵ and γ for a two-dimensional dataset.

Lin-DBSCAN, a machine learning model, is based on a density-based clustering algorithm. This form requires two parameters to be entered: the minimum number of points within the neighborhood (MinPts) and the neighbor radius ε. Both parameters are directly related to the discretization step, which is central to the Lin-DBSCAN approach for network generation. In other words, the parameters Eps, which represents the neighborhood radius, and MinPts, which determine the minimum points within this radius, are intrinsically related to the discretization step within Lin-DBSCAN [9,30]. Lin-DBSCAN indexes the entry points using a grid before evaluating the density-dependent connected areas, where the subdivisions of this grid are aligned with the uniform discretization step. Unlike DBSCAN, which examines each point and its neighborhood, Lin-DBSCAN identifies connected areas based on density by examining grid cells directly. It subdivides this hyper-rectangle uniformly by superimposing a multidimensional grid, the step of which coincides with the selected discretization step. Figure 1 provides a graphical representation of the relationship between the neighborhood radius ε and the discretization step γ in the context of the Lin-DBSCAN clustering algorithm for a two-dimensional dataset. The symbol ε denotes the Eps parameter inherited from DBSCAN, representing the maximum radius within which the algorithm searches for neighboring points to form a cluster. The symbol γ corresponds to the uniform grid cell size used in Lin-DBSCAN, which defines the discretization step applied to the multidimensional space.

As shown in the Figure 1, the grid partitions the space into square cells, and the cells of the grid have a square shape with the length of the edge equal to γ. This discretization step is mathematically defined as

(2) $$\gamma =\frac{\varepsilon }{2\cdot \sqrt{2}}$$

This condition ensures that all points within the same grid cell are located within a distance smaller than ε, guaranteeing their potential connectivity in the density-based clustering process.

The shaded polygonal areas in the figure illustrate dense regions where the number of points (cell cardinality) meets or exceeds the MinPts threshold, indicating candidate clusters. This visual aid reinforces the core principle of Lin-DBSCAN: the efficient identification of dense areas through grid-based indexing rather than point-wise neighborhood evaluation, thus improving computational efficiency while preserving clustering accuracy.

In this study, we propose a novel CFAR processor, Lin-DBSCAN-CFAR, for detecting multiple targets in K-distributed sea clutter. The processor incorporates the Lin-DBSCAN algorithm to identify potential targets and sea spikes, which are often statistical outliers, by separating them from the background clutter. Lin-DBSCAN-CFAR maintains the core framework of traditional CFAR processors but enhances it by including outlier rejection in the detection process. The in-phase and quadrature components of radar returns are utilized to detect outliers using the Lin-DBSCAN algorithm, ensuring that signals classified as outliers maintain their status even after square-law detection. Another notable development involves calculating the threshold factor *a* based on an estimated shape parameter *v*, utilizing a trained Artificial Neural Network (ANN) model and a predefined false alarm probability. Another significant advancement is the derivation of the threshold factor from an estimated shape parameter *v*, using a trained ANN model and a set probability of false alarm. This method contrasts with the closed-form solution employed in conventional CFAR processors designed for Rayleigh-distributed clutter. By removing outliers from reference windows, Lin-DBSCAN-CFAR achieves greater precision in estimating the clutter background level, leading to improved detection performance. In the context of radar signal processing, the complex echo signal received within a reference window can be described as follows:

(3) $$\left[X=\left[x_{1I}+jx_{1Q},x_{2I}+jx_{2Q},\ldots ,x_{NI}+jx_{NQ}\right]\right]$$

**Figure 2:**
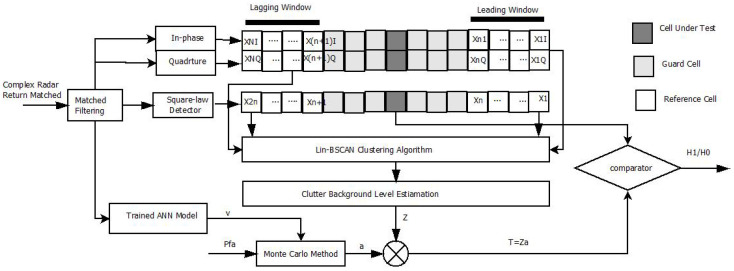
Block diagram of the proposed Lin-DBSCAN-CFAR processor.

Here, $N=2^{n}$ represents the length of the reference window. Additionally, when each range unit sample undergoes processing through a square law detector, the resultant signal is expressed as

(4) $$\left[x_{i}=x_{iI}^{2}+x_{iQ}^{2}\right]$$

where *i* ranges from 1 to *N*. This formulation captures the essential components of the radar’s received signal and its subsequent processing, as shown in Figure 2.

During the detection phase, the Lin-DBSCAN-CFAR processor employs a clustering technique to differentiate between outliers and normal data points within *N* samples of the complex signal *X*. If a specific index in signal *X* is identified as an outlier, then the corresponding sample XM associated with that index in signal Xi is also classified as an outlier. Thus, by recognizing and removing outliers from the Xi and averaging the remaining samples, the background level of clutter in the CUT can be estimated, as explained in reference [3]:

(5) $$Z_{Lin-DBSCAN}=\sum_{i=1}^{N-M}Xi/xm,\;i=1,2\cdots ,N,\;where\;M\in \left[1,N\right]$$

where “*M*” is the number of outliers that have been successfully isolated using the clustering technique.

The decision criteria for the Lin-DBSCAN-CFAR processor are summarized in a binary hypothesis test, concisely stated as follows:

(6) $$X_{0}{≶}_{H_{0}}^{H_{1}}\frac{a}{N-M}\sum_{i=1}^{N-M}Xi/xm,\;i=1,2\cdots ,N,\;where\;M\in \left[1,N\right]$$

In this context, $H_{1}$ represents the presence of a target within the CUT, while $H_{0}$ indicates its absence. In addition, $X_{0}$ and *a* denote the measured value of the CUT and the scaling factor for the threshold, respectively.

The detailed pseudo-code for the Lin-DBSCAN-CFAR processor, incorporating the outlined components, is provided in Algorithm 1.
**Algorithm 1** Proposed Lin-DBSCAN-CFAR Detection Method1:**Input:** Reference cell count: *N*, Guard cell count: *M*, Complex radar return samples in reference window: $\left(x_{1I}+jx_{1Q}\right),\ldots ,\left(x_{NI}+jx_{NQ}\right)$, DBSCAN clustering specifics: Eps and MinPts2:**Output:** Determination of target presence: Either H1 (target detected) or H0 (no target detected).3:**procedure**4:    Initialize empty cluster set: W=∅.5:    Transform each complex radar sample into a 2D point to create dataset $D=\{\left(x_{1I},x_{1Q}\right),\ldots ,\left(x_{NI},x_{NQ}\right)\}$.6:    **for** each point *p* in dataset *D* **do**7:        **if** *p* is already processed **then**8:              Skip to the next point.9:        **else**10:            Evaluate the number of points within Eps-distance of *p*, denote as |NEps(p)|.11:            **if** |NEps(p)| < MinPts **then**12:                 Label *p* as a border point or outlier.13:            **else**14:                 Classify *p* as a core point and assign all points in its Eps-neighborhood to cluster *W*.15:                 **for** each unprocessed point *q* in the Eps-neighborhood of *p* **do**16:                    **if** |NEps(q)|≥ MinPts **then**17:                         Include its neighborhood points in cluster *W*.18:                    **end if**19:                 **end for**20:            **end if**21:         **end if**22:   **end for**23:   Lin-DBSCAN clustering outcome.24:   Compute the clutter level $Z_{Lin-DBSCAN}$ based on Equation (4).25:   Determine target detection (H1 or H0) according to Equation (5).26:**end procedure**

Previous studies indicate that to create smaller clusters and reduce the impact of outliers, the value of MinPts should not be excessively large or small. In general, for two-dimensional data, MinPts=4 is appropriate. Several methods have been proposed to determine the value of the second parameter ε, including equalization histograms and normalized density lists [30]. The value of ε is determined by analyzing the specific features of the sea clutter data [6,25,31]. In general, the optimal value is considered to be ε = 2, as suggested in previous works [24].

## 4. Evaluation and Results

In this part, we deeply scrutinize and compare the SO, GO, OS, CA, DBSCAN, and Lin-DBSCAN-CFAR processors, with a specific focus on understanding the ramifications of interference in multi-target scenarios.

This section includes comprehensive simulations that demonstrate the efficiency and superiority of the Lin-DBSCAN-CFAR processor within sea clutter environments and multiple-target environments. The detection capabilities of the CFAR processor were evaluated and compared with SO, GO, CA, OS, and DBSCAN-CFAR, across an extensive variety of shape parameters, false alarm probabilities, and multi-target scenarios. The simulations were performed on a Windows 10 64-bit system equipped with a 2.40 GHz Intel Core i7 processor and 6 GB of RAM, utilizing MATLAB 2019a for execution.

### 4.1. Impact of Interference in Multi-Target Scenarios

In our research, we strategically placed interference targets within reference cells, centering the primary target in the CUT for detailed analysis. These interference targets were assumed to have the same strength as the primary target. We set the shape parameter *v* to 2.02, following the guidelines of reference [3].

Our comparative study focused on Lin-DBSCAN-CFAR and DBSCAN-CFAR, adhering to the parameters from [3]: 64 reference cells (N) and 4 guard cells (M), ε = 2, MinPts = 4 for both Lin-DBSCAN and DBSCAN-CFAR, a k value of 60 for OS-CFAR, and a false alarm probability ($P_{fa}$) of $P_{fa}=10^{-4}$.

We conducted extensive simulations, running $10^{5}$ Monte Carlo iterations across various SNR for each CFAR processor.

**Figure 3:**
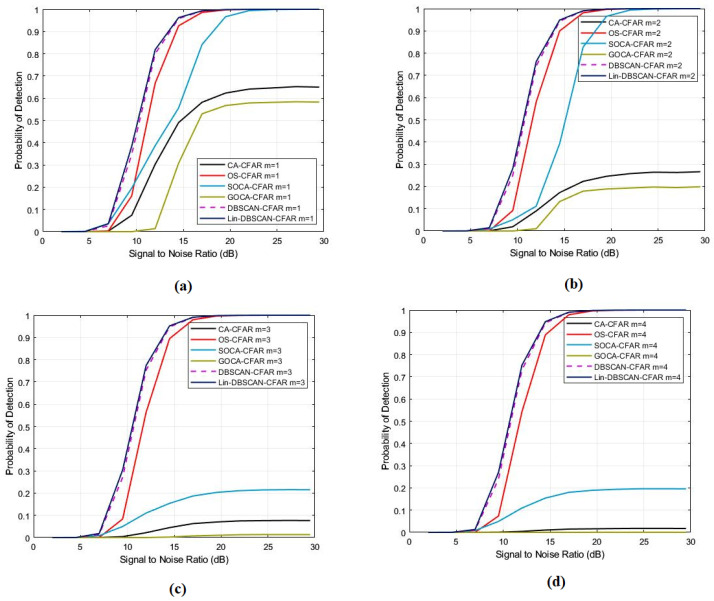
A comparison of the probabilities of detection with different numbers of interference targets for CA, SO, GO, OS, DBSCAN, and Lin-DBSCAN-CFAR (**a**) m = 1, (**b**) m = 2, (**c**) m = 3, (**d**) m = 4.

The Figure 3 illustrates the detection probability across various SNR levels with different counts of interference targets while keeping the probabilities of false alarm and the shape parameters constant. It elucidates the robustness of the Lin-DBSCAN-CFAR and DBSCAN-CFAR algorithms in maintaining high detection probabilities despite the increase in interference targets. This analysis underscores their efficiency in filtering out such interference as extraneous, without relying on prior knowledge of their presence. Notably, both algorithms demonstrated parallel detection performance, highlighting their resilience. In contrast, traditional CFAR processors, such as CA, GO, SO, and OS-CFAR, generally showed a decrease in detection probability with an increase in interference targets. The exception is OS-CFAR, which uniquely preserved its effectiveness. This comprehensive comparison underlines the superior adaptability of Lin-DBSCAN-CFAR amidst a backdrop of high interference targets. It achieves comparable performance to DBSCAN-CFAR with significantly less complexity, marking a significant advancement in the field of target detection methodologies.

### 4.2. Effects of Different Shape Parameters

In our study, we focus on the performance of CFAR processors, using Monte Carlo simulations to examine their detection capabilities. The study particularly explores the impact of varying shape parameters on detection efficiency, while maintaining a constant probability of false alarms ($P_{fa}$). The simulations are conducted with specific settings: overlaps *m* at 5, $P_{fa}$ at $P_{fa}=10^{-4}$, and using N = 64 and M = 4.

The central aspect of our research is to understand how these shape parameters influence CFAR detection in different sea clutter environments. We analyze shape parameters valued at v=0.201,0.502,1.023,6.027,10.109, and 20.121. The aim is to evaluate the impact of these parameters on the performance of CFAR detection processors and their adaptability in various sea clutter scenarios.

**Figure 4:**
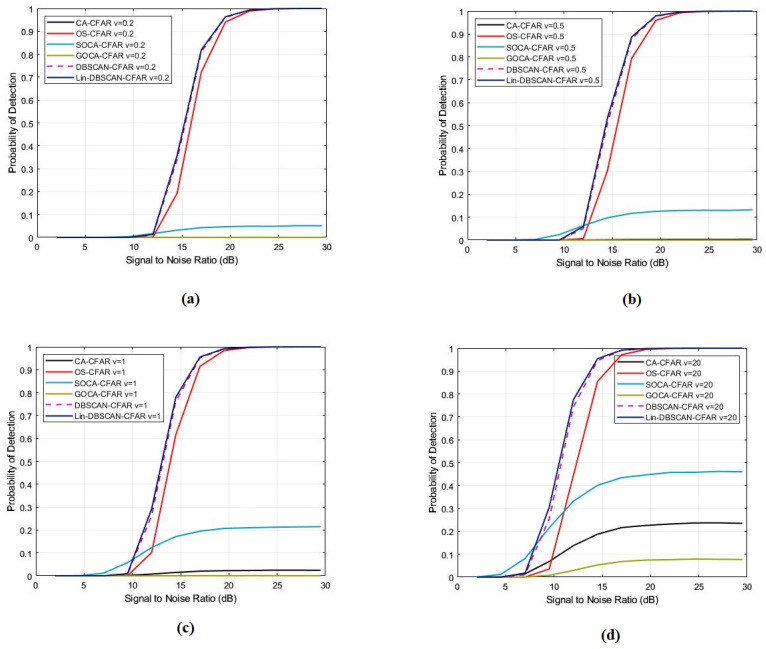
Probability of detection with a different number of ship parameters for CA, SO, GO, OS, DBSCAN, and Lin-DBSCAN-CFAR; (**a**) v = 0.2, (**b**) v = 0.5, (**c**) v = 1, (**d**) v = 20.

In our comprehensive study, as shown in Figure 4, we observed that detection performance significantly improves with the elevation of shape parameters. Particularly, when a shape parameter falls below one, there is a pronounced decline in detection probability due to increased sea peaks, a phenomenon evident in the performance of both OS, Lin-DBSCAN and DBSCAN-CFAR algorithms. Extending our analysis to include CA, SO, and GO-CFAR algorithms, we discovered that their detection probabilities nearly vanish when the shape parameter is below one. Nonetheless, a remarkable enhancement in performance is witnessed as the parameter surpasses this threshold. Among these findings, the Lin-DBSCAN-CFAR method stands out, demonstrating unparalleled detection probability across all examined shape parameter settings. This unified analysis not only highlights the critical role of shape parameters in optimizing detection algorithms but also establishes the Lin-DBSCAN-CFAR method’s superior efficacy under varied conditions, thereby offering a robust framework for enhancing detection capabilities in complex environments.

### 4.3. The Effect of the Probability of a False Alarm

In this comprehensive analysis, we scrutinize the efficacy of various CFAR processors, focusing particularly on their reliability and durability under different false alarm probabilities. The study encompasses four distinct false alarm probabilities: $P_{fa}=10^{-2}$, $P_{fa}=10^{-3}$, $P_{fa}=10^{-4}$, and $P_{fa}=10^{-5}$. Throughout this analysis, we maintain consistent parameters including the number of interfering targets (m = 5) and system parameters (N = 64, M = 4), along with specific values for the shape parameter.

**Figure 5:**
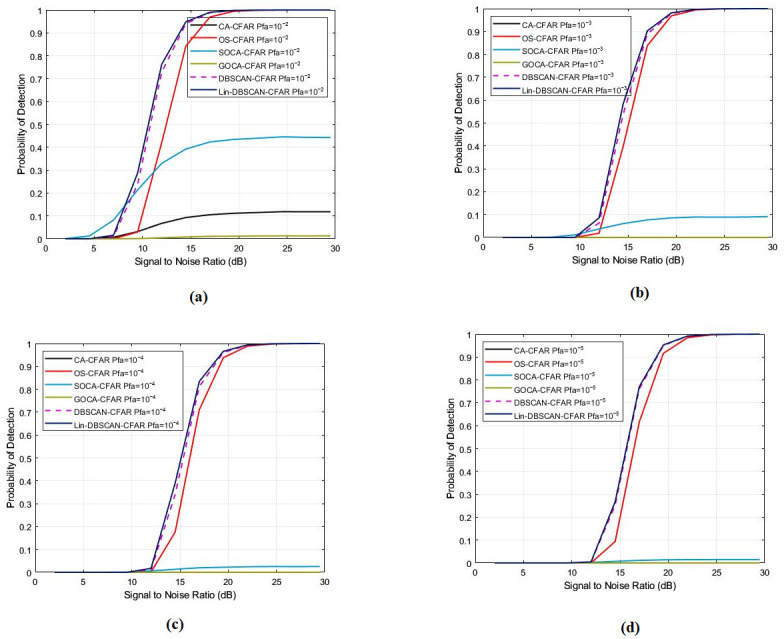
Probability of detection with a different number of false alarm probability for CA, SO, GO, OS, DBSCAN, and Lin-DBSCAN-CFAR; (**a**) $P_{fa}=10^{-2}$, (**b**) $P_{fa}=10^{-3}$, (**c**) $P_{fa}=10^{-4}$, (**d**) $P_{fa}=10^{-5}$.

The data presented in the Figure 5 offer a comprehensive analysis of the performance dynamics of CFAR processors, specifically focusing on DBSCAN-CFAR and Lin-DBSCAN-CFAR, under various conditions of false alarms and SNR levels. Figure 5 demonstrates a clear trend where the probability of detection for both DBSCAN-CFAR and Lin-DBSCAN-CFAR processors increases with the SNR, which ranges from 5 to 30 dB in each simulated scenario. This trend highlights a direct correlation between the enhancement of SNR and the detection capabilities. However, the figure also reveals an unexpected pattern where a decrease in the probability of false alarms correlates with a reduction in detection performance. Despite this, it’s essential to note that both Lin-DBSCAN-CFAR and DBSCAN-CFAR processors consistently outperform their counterparts across all tested false alarm probabilities, emphasizing their superior reliability and robustness. This analysis solidifies the Lin-DBSCAN-CFAR and DBSCAN-CFAR processors as resilient and dependable solutions in the realm of detection applications, showcasing their effectiveness in enhancing detection accuracy while efficiently managing false alarms.

### 4.4. Comparison of SNR Requirements Between OS-CFAR and Lin-DBSCAN-CFAR at $P_{d}=0.8$

In this section, we present a comparison between OS-CFAR and the proposed Lin-DBSCAN-CFAR, as OS-CFAR demonstrates the closest performance to Lin-DBSCAN among traditional CFAR techniques. It is noteworthy that the proposed method achieves detection performance comparable to the more computationally intensive DBSCAN-CFAR while significantly reducing computational complexity. Table 1 presents the required Signal-to-Noise Ratio (SNR) values, in decibels (dB), for both the OS-CFAR and the proposed Lin-DBSCAN-CFAR algorithms to achieve a fixed detection probability of $P_{d}$ = 0.8 under various simulation settings. These settings include different levels of interference target overlap (denoted by *m*), clutter shape parameters (*v*), and constant probabilities of false alarm ($P_{fa}$). From the simulation results, it is evident that the proposed Lin-DBSCAN-CFAR achieves detection performance comparable to the higher-complexity DBSCAN-CFAR algorithm. Notably, Lin-DBSCAN-CFAR consistently requires a 1 to 2 dB lower SNR than the OS-CFAR across all configurations to maintain the same probability of detection. This performance gain is achieved while also benefiting from reduced computational complexity due to Lin-DBSCAN’s efficient grid-based clustering mechanism. The results validate the robustness and efficiency of the proposed technique in environments with varying interference and clutter conditions.

### 4.5. Analysis of Computational Complexity

The complexity of the process in DBSCAN is $O(n^{2})$, which has led to the emergence of numerous algorithms aimed at enhancing its execution time. These include FDBSCAN [31], IDBSCAN [32], HDBSCAN [33], TI-DBSCAN [34], and Grid-DBSCAN [35]. Although these proposed algorithms do enhance execution speed in most cases, the best achievable process complexity is still O(nlogn). As a response to this challenge, Lin-DBSCAN is proposed to further improve process complexity.

The Lin-DBSCAN algorithm checks each data point in the input set once to construct and fill the grid. It computes indexes for the cell to which each data point belongs in each iteration, checking if a non-empty cell already exists in the hash map of the grid. The total cost of this operation is O(n), where n is the total number of data points in the set. This cost efficiency is achieved because the hash map keeps the average access cost of a cell constant. In the collection phase, the Lin-DBSCAN algorithm accesses each cell only once. Therefore, the computational cost of the filling procedure is related to the number of non-empty cells in the grid, denoted by C. This quantity is normally proportional to the size and distribution of cells in the graph. Since the segmentation map ignores the empty cells, they are ignored. As a result, the overall computational complexity is reduced. For the algorithm $O\left(n\right)+O\left(3^{d}\times C\right)$, the optimal scenario will lead to a total cost of O(n)+O(1), since all the input points are contained within a single cell. Conversely, in the worst-case scenario, each entry point is isolated to an odd cell, resulting in a total number of cells equal to the number of points [9]. Here, the total cost becomes O(n)+O(3d×n), where d represents the dimensionality of the data set and 3d signifies the number of cells within a neighborhood. From this analysis, it is evident that Lin-DBSCAN enhances the execution complexity more effectively than DBSCAN and other proposed algorithms.

## 5. Conclusions

This paper proposed the implementation of the Lin-DBSCAN-CFAR processor as a solution to reduce background level estimation variance in K-distributed sea clutter in scenarios involving multiple targets. Lin-DBSCAN clustering technology was utilized to remove outliers, such as sea spikes and interference targets, from the reference window. The background clutter level was estimated using the remaining reference cells. The detection threshold, derived by multiplying the threshold factor by the background clutter level, was then compared with the CUT. Simulations conducted in this study demonstrated that the Lin-DBSCAN-CFAR processor outperformed other tested CFAR processors across different false alarm probabilities, target counts, and shape parameters. Additionally, the simulation results showed that Lin-DBSCAN-CFAR exhibited reduced computational complexity and cost compared with DBSCAN-CFAR. This implies that the utilization of Lin-DBSCAN-CFAR provided advantages in terms of resource efficiency and process effectiveness. These findings further strengthened the effectiveness of Lin-DBSCAN-CFAR as a powerful and reliable tool for addressing challenges related to background level estimation and target discrimination in multi-target scenarios. Moreover, these results served as a valuable foundation for future research endeavors aimed at optimizing and advancing the Lin-DBSCAN-CFAR approach.

## Figures and Tables

**Table 1:** Required SNR (dB) for OS and Lin-DBSCAN CFAR to achieve $P_{d}=0.8$.

Technique	m=1	m=2	m=3	m=4	v=0.2	v=0.5	v=1	v=20	$P_{\mathrm{fa}}=10^{-2}$	$P_{\mathrm{fa}}=10^{-3}$	$P_{\mathrm{fa}}=10^{-4}$	$P_{\mathrm{fa}}=10^{-5}$
OS	13.2 dB	13.8 dB	13.9 dB	14 dB	17.8 dB	17 dB	16 dB	13.8 dB	14.1 dB	16.3 dB	18 dB	18.6 dB
Lin-DBSCAN	12 dB	12.5 dB	12.6 dB	13 dB	16.8 dB	16.1 dB	15 dB	12 dB	12.4 dB	15.8 dB	17 dB	17.3 dB

## Data Availability

Data are contained within the article.

## References

[1] Angelliaume S., Rosenberg L., Ritchie M. (2019). Modeling the amplitude distribution of radar sea clutter. Remote Sens..

[2] Zattouta B., Messikh L. (2018). An adaptive CFAR processor based on automatic censoring technique for target detection in heterogeneous environments. Int. J. Appl. Eng. Res..

[3] Zhao J., Jiang R., Wang X., Gao H. (2019). Robust CFAR detection for multiple targets in k-distributed sea clutter based on machine learning. Symmetry.

[4] Shi S.-N., Shui P.-L. (2016). Optimum coherent detection in homogeneous k-distributed clutter. IET Radar Sonar Navig..

[5] Armstrong B.C., Griffiths H.D. (1991). CFAR detection of fluctuating targets in spatially correlated k-distributed clutter. IEE Proc. F-Radar Signal Process..

[6] Chen X., Liu W., Qiu H., Lai J. (2011). Apscan: A parameter free algorithm for clustering. Pattern Recognit. Lett..

[7] Pan J., Ye S., Shi C., Yan K., Liu X., Ni Z., Yang G., Fang G. (2021). 3D imaging of moving targets for ultra-wideband MIMO through-wall radar system. IET Radar Sonar Navig..

[8] El-Mashade M.B. (2021). Inhomogeneous analysis of novel model of CFAR approaches to detect two-degrees of freedom partially-correlated *χ*^2^-targets. WSEAS Trans. Commun..

[9] Pirrone R., Cannella V., Monteleone S., Giordano G. (2018). Linear density-based clustering with a discrete density model. arXiv.

[10] Rohling H. (1983). Radar CFAR thresholding in clutter and multiple target situations. IEEE Trans. Aerosp. Electron. Syst..

[11] Gandhi P.P., Kassam S.A. (1988). Analysis of CFAR processors in nonhomogeneous background. IEEE Trans. Aerosp. Electron. Syst..

[12] Hansen V.G., Sawyers J.H. (1980). Detectability loss due to “greatest of” selection in a cell-averaging CFAR. IEEE Trans. Aerosp. Electron. Syst..

[13] Gálvez N.B., Cousseau J.E., Pasciaroni J.L., Agamennoni O.E. (2012). Improved neural network based CFAR detection for non-homogeneous background and multiple target situations. Lat. Am. Appl. Res..

[14] Wang C., Guo B., Song J., He F., Li C. (2024). A novel CFAR-based ship detection method using range-compressed data for spaceborne SAR system. IEEE Trans. Geosci. Remote Sens..

[15] Zhang Q., Zhu Y., Cordeiro F.R., Chen Q. (2025). PSSCL: A progressive sample selection framework with contrastive loss designed for noisy labels. Pattern Recognit..

[16] Zhang Q., Jin G., Zhu Y., Wei H., Chen Q. (2024). BPT-PLR: A balanced partitioning and training framework with pseudo-label relaxed contrastive loss for noisy label learning. Entropy.

[17] Zhang Q., Zhu Y., Yang M., Jin G., Zhu Y.W., Chen Q. (2024). Cross-to-merge training with class balance strategy for learning with noisy labels. Expert Syst. Appl..

[18] Liu W., Liu J., Liu T., Chen H., Wang Y.-L. (2023). Detector design and performance analysis for target detection in subspace interference. IEEE Signal Process. Lett..

[19] Liu W., Liu J., Hao C., Gao Y., Wang Y.-L. (2022). Multichannel adaptive signal detection: Basic theory and literature review. Sci. China Inf. Sci..

[20] Rihan M.Y., Nossair Z.B., Mubarak R.I. (2024). An improved CFAR algorithm for multiple environmental conditions. Signal Image Video Process..

[21] Al-dabaa M.M., Emran A.A., Yahya A., Aboshosha A. (2024). Deep Learning Mitigation of Sea Clutter for Enhanced Radar Target Detection. J. Al-Azhar Univ. Eng. Sect..

[22] AlTuwaim B.N. (2023). A Metaheuristic to Solve the Capacitated Clustering Problem. J. Al-Azhar Univ. Eng. Sect..

[23] Hamza K.S., Amir F.A. (2017). Evolutionary Algorithms for Clustering in Wireless Sensor Networks. J. Al-Azhar Univ. Eng. Sect..

[24] Bryant A., Cios K.R. (2017). RNN-DBSCAN: A density-based clustering algorithm using reverse nearest neighbor density estimates. IEEE Trans. Knowl. Data Eng..

[25] Zhou W., Xie J., Xi K., Du Y. (2018). Modified cell averaging CFAR detector based on Grubbs criterion in non-homogeneous background. IET Radar Sonar Navig..

[26] Mou X., Chen X., Guan J., Chen B., Dong Y. Marine target detection based on improved Faster R-CNN for navigation radar PPI images. Proceedings of the 2019 International Conference on Control, Automation and Information Sciences (ICCAIS).

[27] Ji Y., Liu A., Shao S., Yu C., Chen X. (2025). Intelligent Target Detection Method for HFSWR based on Dual-scale Branch Fusion Network and Adaptive Threshold Control. IEEE Trans. Radar Syst..

[28] Li Y., Zhu J., Zhang J., Wang W., Duan C. (2019). Two-step detection algorithm for fluctuating weak target based on dynamic programming. J. Eng. Technol. (JET).

[29] Wang R., Li J., Duan Y., Cao H., Zhao Y. (2018). Study on the combined application of CFAR and deep learning in ship detection. J. Indian Soc. Remote Sens..

[30] Hou J., Gao H., Li X. (2016). DSETS-DBSCAN: A parameter-free clustering algorithm. IEEE Trans. Image Process..

[31] Amini A., Saboohi H., Wah T.Y., Herawan T. (2014). A fast density-based clustering algorithm for real-time internet of things stream. Sci. World J..

[32] Borah B., Bhattacharyya D.K. An improved sampling-based DBSCAN for large spatial databases. Proceedings of the International Conference on Intelligent Sensing and Information Processing.

[33] Campello R.J.G.B., Moulavi D., Zimek A., Sander J. (2015). Hierarchical density estimates for data clustering, visualization, and outlier detection. ACM Trans. Knowl. Discov. Data (TKDD).

[34] Kryszkiewicz M., Lasek P. TI-DBSCAN: Clustering with DBSCAN by means of the triangle inequality. Proceedings of the International Conference on Rough Sets and Current Trends in Computing.

[35] Mahran S., Mahar K. Using grid for accelerating density-based clustering. Proceedings of the 8th IEEE International Conference on Computer and Information Technology.

